# Editorial: Transcription Regulation—Brain Development and Homeostasis—A Finely Tuned and Orchestrated Scenario in Physiology and Pathology

**DOI:** 10.3389/fnmol.2021.834607

**Published:** 2022-01-14

**Authors:** Estela M. Muñoz, Flavio S. J. de Souza, Martin F. Rath, Verónica Martínez Cerdeño

**Affiliations:** ^1^Institute of Histology and Embryology of Mendoza (IHEM), National University of Cuyo (UNCuyo), National Scientific and Technical Research Council (CONICET), Mendoza, Argentina; ^2^Institute of Physiology, Molecular Biology and Neurosciences (IFIBYNE), National Scientific and Technical Research Council (CONICET) and Department of Physiology, Molecular and Cellular Biology, Faculty of Exact and Natural Sciences, University of Buenos Aires (FBMC-FCEN-UBA), Buenos Aires, Argentina; ^3^Department of Neuroscience, Faculty of Health and Medical Sciences, University of Copenhagen, Copenhagen, Denmark; ^4^Department of Pathology and Laboratory Medicine, UC Davis School of Medicine, Institute for Pediatric Regenerative Medicine, Shriners Hospitals for Children of Northern California, and MIND Institute at the UC Davis Medical Center, Sacramento, CA, United States

**Keywords:** brain, transcription, transcription factor, neurogenesis, development, neurodevelopmental disorders, neurodegenerative diseases

A finely tuned regulation of gene expression is essential for shaping the nervous system and for maintaining its homeostasis throughout life. Disruptions in gene regulation can impact brain development and physiology in ways that contribute to diverse pathologies. Classic and state-of-the art experimental models and technologies have advanced our knowledge of transcriptional regulators and the ways they interact in the healthy and diseased brain. Further in-depth characterization of the mechanisms of transcriptional regulation is needed to better understand how each element, from genes to cells, defines and maintains identities and functionalities in the nervous system. This Research Topic focuses on transcriptional regulation within the nervous system, with an emphasis on developmental and homeostatic processes, their dysregulation, and their association with neurodevelopmental disorders and neurodegenerative diseases. Eleven peer-reviewed manuscripts including six original articles, three reviews, one mini review, and one brief research report, encompass this special volume. Fifty-nine authors from research laboratories located in 10 countries: Argentina, Canada, China, Germany, Israel, Russia, Serbia, United Kingdom, United States, and Vietnam, took part in this initiative.

Among the interesting contributions, Oproescu et al. beautifully reviewed the regulatory intricacies of the proneuronal basic-helix-loop-helix (bHLH) transcription factors (TFs) in the cell quiescence-to-proliferation-to-differentiation continuum within the murine cerebral cortex, drawing parallels with other organisms and neural tissues. The authors discuss diverse mechanisms that govern bHLH TF expression, stability, localization, and consequent transactivation of downstream target genes, in a temporally defined and context-dependent manner. The authors conclude that further in-depth understanding of bHLH TF complexity and interactions might be useful to improve neuronal reprogramming strategies for regenerative medicine purposes.

Within the bHLH superfamily, closely related Neurod1, Neurod2, Neurod4 and Neurod6 have emerged as essential TFs for the correct functioning of cells during development and postnatal life. The Neurod family was reviewed by Tutukova et al. in the context of the developing and mature cerebral cortex, and other areas of the central nervous system such as the cerebellum, the brainstem, and the spinal cord. bHLH TFs are presented as both ubiquitous and cell-specific regulators responsible for a variety of biological functions, that range from progenitor cell proliferation and survival to neuronal differentiation, neuronal migration, fate specification, axonal navigation, dendritic elongation, and synaptic formation. The authors also present recent links between Neurod dysfunction and neurological disorders in humans, such as Alzheimer's disease, and they also discuss the Neurod family's potential use as biomarkers.

Stevanovic et al. summarize current knowledge of the roles of SOX proteins in controlling nervous system development and homeostasis in normal and pathological conditions. The SOX TFs are presented herein as a multifaceted superfamily involved in maintaining stem cell pluripotency and paradoxically, during processes that determine neuronal and glial differentiation in the developing brain. They act as both pioneer TFs and sequential regulators. SOX proteins are also key regulators of adult neurogenesis. The authors discuss regulatory mechanisms of SOX functions and the deleterious effects of deregulation of specific SOX genes in the context of neurodevelopmental disorders and neurodegenerative diseases.

Tbr2 (Eomes) is a T-box TF expressed in intermediate progenitors of the developing cerebral cortex and can be used as a molecular marker to label these cells. Bedogni and Hevner combined transcriptome profiling of cell types in the embryonic cerebral cortex of transgenic *Tbr2-GFP* mice with *in situ* hybridization, to identify genes whose expression is enriched in intermediate progenitors. They found that intermediate progenitors not only amplify neurogenesis quantitatively but also, they molecularly “prime” new projection neurons for axogenesis, guidance, and intrinsic excitability. These novel functions make intermediate precursors active participants in optimizing axon development and integration into cortical pathways, in addition to their known roles in shaping regional and laminar identity of projection neurons, and in signaling to interneurons and radial glial progenitors.

Neuronal migration is a critical process in cortical layer formation, and its dysregulation can lead to cerebral cortex malformations and neurodevelopmental disorders. The study authored by Sokpor et al., highlights the indispensable role of the ATP-dependent chromatin remodeling BAF complex in the formation of cortical layers during mouse development. This mechanism regulates multiple aspects of radial neuronal migration under the influence of Wingless/Int (WNT) signaling. Using three different mouse models, the authors applied cellular and molecular analyses of the developing cerebral cortex after temporally and cell-type specifically inactivating components of the BAF complex.

Goodwin et al. contributed to this collection with an original research article that links chromatin remodeling with neuron differentiation and brain size. The authors utilized cerebellar granule neuron precursor cultures from *Smarca1* mutant mice (Ex6DEL) to explore the influence that the nucleosome-remodeling factor subunit Snf2l has on progenitor homeostasis. They show that Ex6DEL progenitors have a transient delay in cell cycle withdrawal, but ultimately these cells do differentiate. This may account for the increased brain size in Ex6DEL mutant mice. Interestingly, Ex6DEL progenitors have a more relaxed chromatin configuration at several regions of the genome which are enriched in binding sites for Fos/Jun TFs.

MiR-184 is a highly enriched microRNA in the mammalian brain and one of the key downregulated circulating microRNAs in patients following ischemic stroke. Yang et al. show that miR-184 is crucial to alleviate damage in an ischemic stroke rat model and, in an oxygen-glucose deprivation/reoxygenation cell model. The authors propose that the targeting of the phosphatidic acid phosphatase type 2B (PPAP2B) mRNA is part of the protective molecular mechanism elicited by miR-184, and therefore they suggest that it might be a promising therapeutic option for stroke recovery.

Regarding neuronal differentiation, Sampieri et al. show that CREB3L2, a member of the CREB3 TF family, is an inhibitory downstream effector during nerve growth factor (NGF)-induced PC12 cell differentiation. CREB3L2 expression is increased upon NGF-induced differentiation, being a target shared by PKA and MAPK/ERK signaling pathways. CREB3L2 enhances Rab5 GTPase levels, a negative regulator of neuronal differentiation, and it also inhibits NGF-induced neurite growth. The authors conclude that finely tuned modulation of CREB3L2 seems to be necessary for PC12 differentiation triggered by NGF.

MANF (Mesencephalic astrocyte-derived neurotrophic factor) belongs to a novel family of secreted neurotrophic regulators. By using cell culture models in which MANF expression was altered, Wen et al. show that this factor regulates neurite outgrowth by activating Akt/mTOR and Erk/mTOR signaling pathways and protein synthesis. The authors propose that MANF may be a potential candidate to facilitate the regeneration of neuronal processes in neurodegenerative diseases.

Autism spectrum disorder is among the many neurodevelopmental disorders that show sex differences. The involved mechanisms are yet unclear, although members of the large family of nuclear receptor TFs appear to be potential candidates. In their mini review, Arnold and Saijo discuss the potential role of sex steroid hormones and their receptors, especially the estrogen receptor β, in a mouse model of maternal immune activation that is commonly used to study autism spectrum disorder. The authors hypothesize that estrogen receptor β-mediated repression of inflammation in brain myeloid-lineage cells may contribute to the male bias that they observed in the maternal immune activation model. Also, they propose new avenues of research on this subject.

Circadian clocks, as they have evolved, are excellent prototypes for studying how cellular transcription is linked to external signal. Functional circadian clocks imply interlocking transcription-translational feedback loops at the cellular level that directly or indirectly respond to external cues such as light information. Clock genes and clock-controlled genes are key participants; among them, the *per* family. In an original research article, Ruggiero et al. show the consequences of a loss-of-function *per2* gene mutation in maintaining rhythmic expression of circadian clock genes, as well as clock-controlled genes, which consequently affects the rhythmic behavior of intact zebrafish larvae. Furthermore, they demonstrate that disruption of the *per2* gene impacts on the circadian regulation of the cell cycle *in vivo* in a tissue-specific manner.

The original research and review articles of this collection illustrate how transcription factors, chromatin remodelers, microRNAs, secreted factors, and intracellular transduction proteins are all necessary for the harmonious development and functioning of the nervous system. We expect this collection will stimulate the scientific community to continue its efforts to further understand cellular and molecular mechanisms in both healthy and diseased nervous system. Potential new biomarkers and novel treatments might emerge from this and other initiatives.

## Author Contributions

EM, FS, MR, and VM wrote the Research Topic proposal, edited articles, assigned reviewers, wrote the Editorial draft, and edited and approved its final version. EM contacted and invited contributors. All authors contributed to the article and approved the submitted version.

## Funding

EM was supported by grants from ANPCyT (Argentina; PICT 2017-00499; http://www.agencia.mincyt.gob.ar), CONICET (Argentina; PUE 2017; http://www.conicet.gov.ar), NIH-CONICET (Argentina and USA; F65096), and NIH (USA; 2 R01 GM083913-41A1; https://www.nih.gov). FS was supported by grant from ANPCyT (Argentina; PICT 2018-03947). MR was supported by grants from the Lundbeck Foundation (Denmark; R344-2020-261; https://lundbeckfonden.com), the Independent Research Fund Denmark (Denmark; 1030-00045B; https://dff.dk), and the Novo Nordisk Foundation (Denmark; NNF21OC0070214; https://novonordiskfonden.dk). VM was supported by grants from NIMH and NINDS (USA; RO1 MH094681; https://www.nimh.nih.gov; RO1 NS10713; https://www.ninds.nih.gov).

## Conflict of Interest

The authors declare that the research was conducted in the absence of any commercial or financial relationships that could be construed as a potential conflict of interest.

## Publisher's Note

All claims expressed in this article are solely those of the authors and do not necessarily represent those of their affiliated organizations, or those of the publisher, the editors and the reviewers. Any product that may be evaluated in this article, or claim that may be made by its manufacturer, is not guaranteed or endorsed by the publisher.

